# Prognostic values of novel biomarkers in patients with AL amyloidosis

**DOI:** 10.1038/s41598-019-48513-6

**Published:** 2019-08-21

**Authors:** Darae Kim, Ga Yeon Lee, Jin-Oh Choi, Kihyun Kim, Seok Jin Kim, Eun-Seon Ju, Eun-Seok Jeon

**Affiliations:** 1 Division of Cardiology, Department of Medicine, Heart Vascular Stroke Institute, Samsung Medical Center, Sungkyunkwan University School of Medicine, Seoul, Republic of Korea; 2 Divsion of Hematology and Oncology, Department of Medicine, Samsung Medical Center, Sungkyunkwan University School of Medicine, Seoul, Republic of Korea

**Keywords:** Haematological diseases, Predictive markers

## Abstract

As cardiac involvement is the most important prognostic marker in light-chain amyloidosis (AL), revised Mayo staging for AL incorporated N-terminal pro-brain natriuretic peptide (NTproBNP) and troponin T (TnT). However, prognostic value of novel biomarkers, such as soluble suppression of tumorigenicity 2 (sST2), growth differentiation factor 15 (GDF15), or osteopontin (OPN) is unknown in AL amyloidosis. We aimed to investigate additive predictive effects of novel biomarkers for overall mortality rates of AL amyloidosis patients. Levels of sST2, GDF15, and OPN were quantified at diagnosis in a total of 73 AL amyloidosis patients at Samsung Medical Center from 2010 to 2016. The median follow-up duration of the censored cases was 18.0 (12.4–28.1) months. A total of 25 deaths occurred during the follow-up period. Two novel biomarkers, sST2 and GDF-15 showed satisfactory predictive performances for both one-year and overall survival from ROC analysis. Best cut-off values for predicting one-year mortality were selected. Elevated sST2 and GDF-15 levels showed significant incremental prognostic values in addition to NT-ProBNP and TnT for overall mortality. Patients were assigned 1 point for elevated sST2 or GDF-15. The mean values of NT-proBNP, TnT, mean LV wall thickness, and septal e′ velocity differed significantly according to the scores. Patients with higher scores showed significantly worse prognosis even in patients with advanced revised Mayo staging. Two novel biomarkers, sST2 and GDF-15, showed satisfactory prognostic value for overall survival of AL amyloidosis patients. Furthermore, sST2 and GDF-15 showed additive incremental values over conventional biomarkers and further discriminated prognosis of patients in advanced stages.

## Introduction

Cardiac involvement is the most important prognostic marker in light-chain amyloidosis (AL). Revised Mayo staging is the most widely used, well-validated prognostic system in AL amyloidosis, incorporating the cardiac biomarkers troponin T (TnT) and N-terminal pro-brain natriuretic peptide (NT-proBNP)^1^. According to the current staging, patients with cardiac involvement are most likely classified as advanced stage. However, recent advances in chemotherapy regimens for AL amyloidosis have improved overall survival, even in patients with cardiac involvement^2,3^. Furthermore, previous studies have showed that cardiac amyloid load is significantly associated with prognosis of AL amyloid patients, suggesting different prognosis according to degree of cardiac involvement^4^. Therefore, more detailed risk stratification of AL amyloidosis patients with cardiac involvement is needed.

Although NT-proBNP is a very sensitive marker for assessment of cardiac involvement in AL amyloidosis^5^, previous studies have failed to show a correlation between cardiac amyloid load and NT-proBNP in AL amyloidosis^4^. Considering the complex pathophysiology of cardiac involvement in AL amyloidosis, a simple combination of NT-proBNP and TnT may not provide sufficient prognostic information. Novel biomarkers with different pathophysiological targets would contribute to detailed risk stratification, which could facilitate development of risk-adapted therapies in AL amyloidosis patients, especially those with cardiac involvement.

Recently, novel biomarkers have been introduced and their prognostic values have been studied in heart failure patients. The prognostic performance of soluble suppression of tumorigenicity 2 (sST2), growth differentiation factor 15 (GDF-15), and osteopontin (OPN) has been studied in various populations with heart failure^6–8^. In this study, we sought to determine prognostic value of these three novel biomarkers in AL amyloidosis. We also investigated if prognostic values of these biomarkers had additive incremental value over the currently used biomarkers NT-proBNP and TnT.

## Results

### Patient characteristics

Table 1 describes the main characteristics of patients. Forty-four patients (60%) were men and mean age at baseline was 60 years. Kidney involvement was present in 41 (56%) of patients and cardiac involvement was detected in 50 (69%) patients. Thirty-four (47%) patients were classified as Revised Mayo stage IV. A total of 63 (86%) underwent chemotherapy and 10 patients did not receive chemotherapy. A total of 20 (27%) patients underwent autologous stem cell transplantation. Median follow up was 14.2 months (range 0.4–65 months). A total of 25 patients died during follow-up.

**Table 1:** Clinical characteristics of study patients.

No. of patients	N = 73
Age (years)	60 ± 10
Men, n (%)	44 (60)
Lambda light chain, n (%)	56 (76%)
**Amyloid organ involvement, n (%)**	
Heart	50 (68.5)
Kidney	41 (56.2)
Gastrointestinal tract	16 (21.9)
Peripheral nerve	27 (36.9)
**Revised Mayo staging, n (%)**	
I	14 (19.2)
II	12 (16.4)
III	13 (17.8)
IV	34 (46.6)
LA volume index (mL/m^2^)	41.0 ± 13.5
E′ (cm/s)	5.01 ± 1.82
E/E′	19.3 ± 2.00
Mean LV wall thickness (mm)	11.6 ± 0.31
LV ejection fraction (%)	59.3 ± 1.3
NT-proBNP (pg/mL)	2141 (327.2–6746.5)
Troponin T (ng/mL)	0.051 (0.019–0.093)
Creatinine (mg/dL),	0.89 (0.71–1.11)
eGFR (mL/min/1.73 m^2^)	79.6 (58.9–94)
Free light chain difference (mg/mL)	309.0 (133.1–726.9)
Autologous stem cell transplantation, n (%)	20 (27%)

Median (range), mean ± SD

LA, left atrium; LV, left ventricle; eGFR, estimated glomerular filtration rate

### Novel biomarkers and overall survival

**Figure 1:**
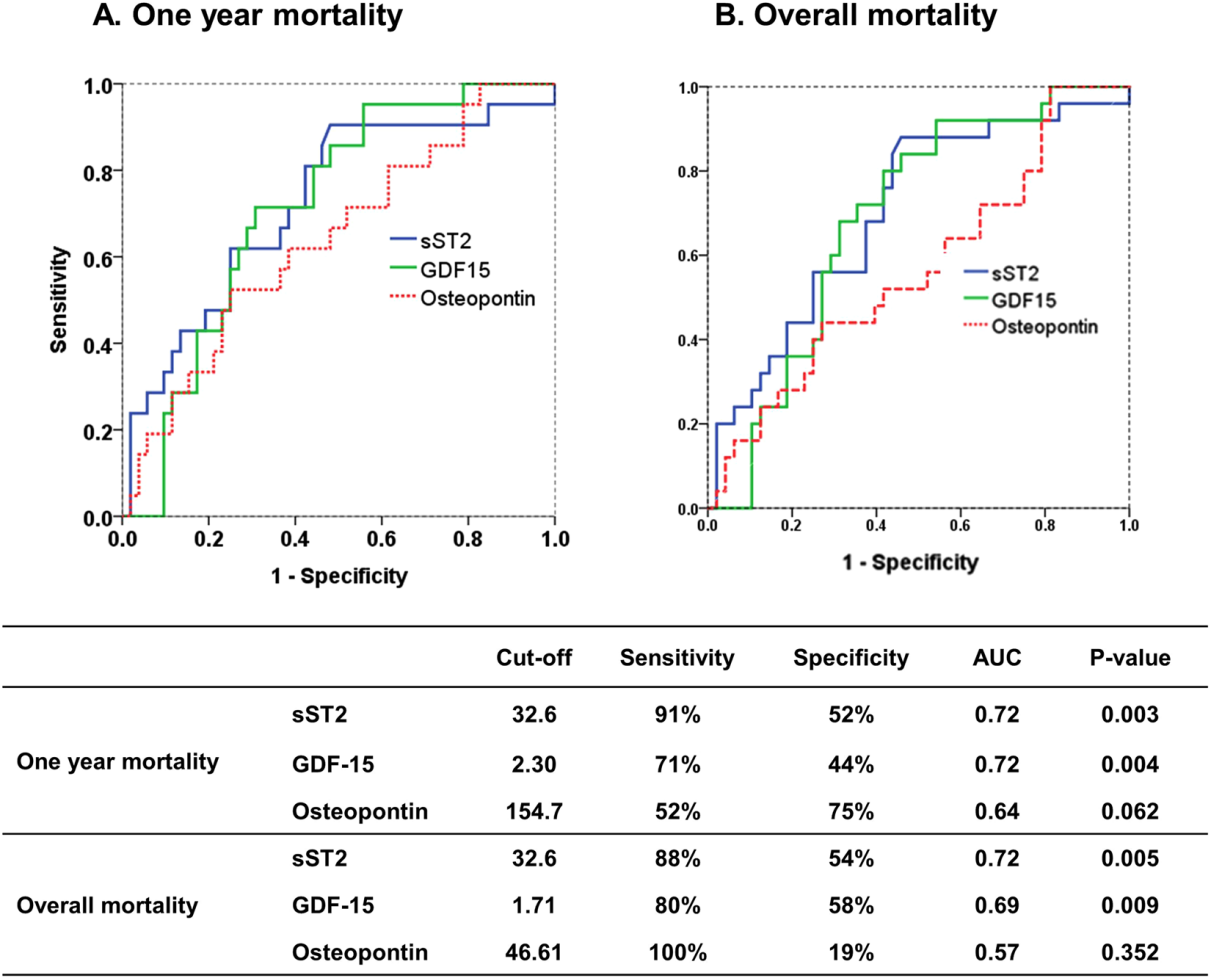
ROC curves of novel biomarkers for predicting one-year (**A**) and overall mortality (**B**).

Median values of sST2, GDF-15, and OPN were as follows: 39.8 ng/mL (IQR 25.5, 70.8), 1.89 ng/mL (IQR 1.04, 3.79), and 107.2 ng/mL (IQR 64.0, 170.0), respectively. There were no age- or sex-related relationships among biomarkers. Receiver operating characteristic (ROC) curves were used to derive cut-off values of novel biomarkers for predicting one-year and overall mortality (Fig. 1). The best cut-off values for sST2 to predict one-year and overall mortality were 32.6 ng/mL (AUC 0.72, sensitivity 91%, specificity 52%, p = 0.003 for one-year survival; AUC 0.72, sensitivity 88%, specificity 54%, p = 0.005 for overall survival). The respective cut-off values for GDF-15 were 2.30 ng/mL (AUC 0.72, sensitivity 71%, specificity 44%, p = 0.004 for one-year mortality) and 1.71 ng/mL (AUC 0.69, sensitivity 80%, specificity 58%, p = 0.009 for overall mortality). OPN did not show significant predictive values for predicting one-year or overall mortality.

**Figure 2:**
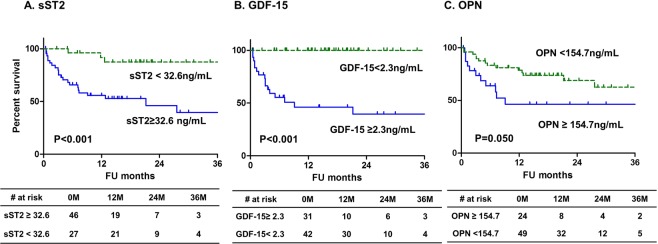
Kaplan survival curves for overall survival according to levels of novel biomarkers.

Elevated levels of sST2, GDF-15, and OPN were defined by cut-off values from previous ROC curves for predicting one-year mortality (sST2 ≥ 32.6 ng/mL, GDF-15 ≥ 2.3 ng/mL, OPN ≥ 154.7 ng/mL). Patients with elevated levels of sST2 and GDF-15 at baseline showed significantly poor overall survival compared to those with lower levels (Fig. 2). Elevated sST2 and elevated GDF-15 showed significant incremental predictive values for overall mortality over elevated traditional biomarkers (NT-proBNP ≥ 1,800 pg/mL and/or TnT ≥ 0.025 ng/mL) in a step-wise manner (Supplemental Fig. 1). From multivariate analysis, elevated sST2 and GDF-15 were independently associated with overall mortality (Table 2).

**Table 2:** Univariate and multivariate analysis for overall mortality.

	Univariate analysis		Multivariate analysis	
	HR (95% CI)	P value	HR (95% CI)	P value
Age, years	1.03 (0.99–1.07)	0.282		
Male sex	1.09 (0.48–2.48)	0.639		
eGFR (mL/min/1.73 m^2^)	1.003 (0.99–1.02)	0.602		
Revised Mayo Stage III/IV	3.89 (1.39–10.83)	0.010	1.89 (0.79–4.52)	0.149
sST2 ≥ 32.6 ng/mL	6.16 (1.84–20.64)	0.003	3.94 (1.12–13.8)	0.033
GDF-15 ≥ 2.3 ng/mL	3.82 (1.68–8.72)	0.001	2.53 (1.08–5.92)	0.032
OPN ≥ 154.7 ng/mL	2.26 (1.02–5.01)	0.144		

eGFR, estimated glomerular filtration rate; sST2, Soluble suppression of tumorigenicity 2; GDF-15, growth differentiation factor 15; OPN, osteopontin.

### Elevated sST2 and GDF-15 in association with conventional biomarkers, LV functions, and prognosis

**Figure 3:**
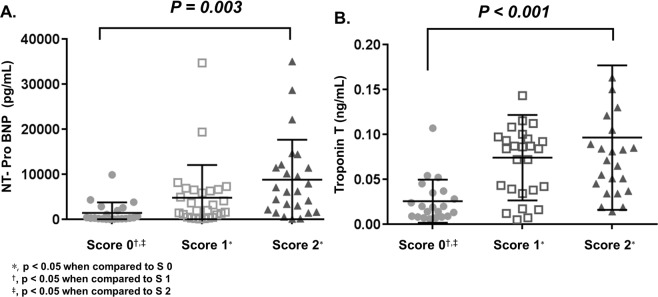
Comparison of mean values of NT-proBNP (**A**) and TnT (**B**) according to scores given by levels of sST2 and GDF-15.

Patients were scored by assigning 1 point for each of elevated sST2 (sST2 ≥ 32.6 ng/mL) and GDF-15 (GDF-15 ≥ 2.3 ng/mL) [Score 0 (neither elevated sST2 nor GDF-15), Score 1 (elevated sST2 or GDF-15), Score 2 (both elevated sST2 and GDF-15)]. Patients were classified into 3 groups according to these scores. Mean values of NT-proBNP and TnT differed significantly among three groups (Fig. 3A,B) and they showed increasing trends with higher scores. Mean values of septal e′ and mean LV wall thickness also differed significantly among three groups (Supplemental Fig. 2A,B). Patients with higher scores had more reduced longitudinal motion of LV (reflected by septal e′ velocity) and relatively more thickened LV wall.

**Figure 4:**
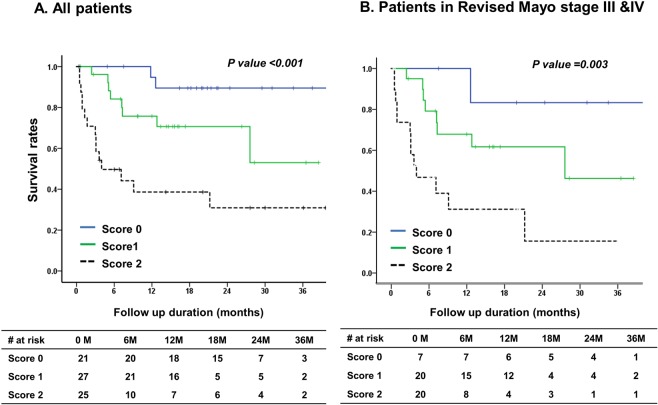
Kaplan-Meier survival curve for overall survival according to scores given by levels of sST2 and GDF-15 in all patients of AL amyloidosis cohorts (**A**) and in subgroups of patients with advanced revised Mayo stages (stage III and IV).

Patients with higher scores showed significantly worse prognosis (Fig. 4A). This trend remained to be significant in subgroups of patients with advanced revised Mayo staging (stage III and IV) (Fig. 4B) or those with cardiac involvement (Supplemental Fig. 3).

## Discussion

In the current analysis, we evaluated prognostic performances of novel biomarkers in patients with AL amyloidosis. The result obtained in this cohort of patients show that (1) the novel biomarkers sST2 and GDF-15 show satisfactory prognostic values for overall survival in patients with AL amyloidosis, (2) sST2 and GDF-15 had incremental prognostic values over the conventional biomarkers NT-proBNP and TnT, and (3) sST2 and GDF-15 further discriminated prognosis of patients at advanced revised Mayo stages.

Recently, new biomarkers have been introduced and validated in heart failure patients to attempt accurate diagnosis and prognostic evaluation. As cardiac involvement is pivotal for prognosis in AL amyloidosis, parameters reflecting cardiac function or pathology are likely to be closely related to prognosis of AL amyloidosis. Almost half of AL amyloidosis patients have cardiac amyloidosis at time of the diagnosis^9^. As survival of AL amyloidosis patients has increased due to the introduction of novel agents and stem cell transplantation, more detailed risk stratification is needed in patients with cardiac amyloidosis, whom are often classified as advanced stages according to revised Mayo staging system. Novel biomarkers also may complement limitations of traditional biomarkers. For example, although prognostic value of NT-proBNP in AL amyloidosis has been recapitulated in previous studies, the value of NT-proBNP is greatly affected by volume status or renal function of patients^5,10^.

sST2 is in the IL-1 receptor family and plays an important role through IL-33 signaling^11^. sST2 is elevated in heart failure patients and acts as decoy receptor of IL-33 to alleviate cardio-protective effects of IL-33^12,13^. Many studies have reported a significant association between sST2 and outcome in chronic and acute heart failure patients^14–16^. GDF-15 is a member of the transforming growth factor-β superfamily and is highly expressed in cardiac myocytes, especially during tissue injury and inflammatory status^17,18^. Prognostic utility of GDF-15, as a biomarker of inflammatory stress, has been well-demonstrated in HF patients with both reduced and preserved ejection fraction^19,20^. OPN is a secreted phosphor-glycoprotein expressed in many cell types including cardiomyocytes^21^. Results from previous studies suggest that OPN is upregulated in animal models of heart failure in response to biomechanical stress. Expression of OPN is elevated and correlated with left ventricular function and diameter in patients with myocardial infarction and dilated cardiomyopathy^22,23^.

From our results, sST2 and GDF-15 showed satisfactory discriminative values for overall survival in AL amyloidosis patients, while OPN did not. A combination of sST2 and GDF-15 showed significant incremental prognostic values over the traditional biomarkers TnT and NT-proBNP. Also, classifications according to levels of sST2 and GDF-15 showed good correlations with structure (mean wall thickness) and longitudinal function (septal e′ velocity) of left ventricle. Our results suggest possible utilization of sST2 and GDF-15 for further discrimination of prognosis of AL amyloid patients who were previously classified as advanced stages. Risk stratification with multi-biomarkers would facilitate selection of patients for new treatment strategies or novel agents.

There are some limitations to our study. The number of patients is relatively small from a statistical standpoint and suggested cut-off values need to be validated in further studies. We acknowledge that this was single-center cohort study and that the prognosis and baseline characteristics of our patients may not reflect the worldwide AL patient population. However, our center is one of largest referral centers for AL amyloidosis in our country. Further, because all studies were carried out in a single center, there was great consistency in blood sampling and measurement of novel biomarkers and all markers were available in all patients. Further study in a larger independent AL amyloidosis population is needed to fully validate and confirm our findings indicating that measurement of sST2 and GDF-15 provides additional prognostic value in AL patients with cardiac involvement.

In conclusion, the novel biomarkers sST 2 and GDF-15 showed satisfactory prognostic values for overall survival in patients with AL amyloidosis. Furthermore, sST2 and GDF-15 showed additive incremental values over conventional biomarkers and further discriminated prognosis of patients in advanced revised Mayo stages (stage III and IV). These new biomarkers may contribute to further detailed risk stratifications in AL amyloidosis, especially with cardiac involvement. Further studies are needed for validation of suggested cut-off values and investigation of the potential use for monitoring treatment effects.

## Materials and Methods

### Patient population

Levels of sST2, GDF-15, and OPN were assessed at time of diagnosis in 73 AL amyloidosis patients at Samsung Medical Center from 2010 to 2015. All patients were biopsy-proven amyloidosis confirmed by Congo red staining and immunohistochemistry of any tissue specimen using commercially available monoclonal antibodies as in previous studies^4^. The diagnosis of AL amyloidosis required tissue confirmation of amyloid deposits or fibrils by apple-green birefringence with Congo red staining, kappa or lamda restriction by immunohistochemistry in at least one involved organ, and evidence of monoclonal gammopathy. Cardiac involvement was defined by the presence of amyloid deposits on endomyocardial biopsy (n = 50, 69%). Patients with dialysis were excluded due to altered metabolism of biomarkers. Plasma cell dyscrasia was documented by serum/urine immunofixation electrophoresis and serum free light-chain test. All patients agreed to the use of blood samples for experimental purposes, in accordance with institutional review board guidelines.

The mortality endpoint was defined as time to death from baseline for all deceased patients or time to censor date, February 28, 2015. Date of baseline was within 1 month of diagnosis. Occurrence and date of death and ongoing survival status were regularly monitored by visits or telephone calls at Samsung Medical Center. The median follow-up duration of the censored cases was 18.0 (12.4–28.1) months.

### Study procedure

Baseline assessment included standardized physical examination, history taking, 12-lead electrocardiogram, transthoracic echocardiography, and blood sampling. Modification of Diet in Renal Disease (MDRD) equation was used to calculate estimated glomerular filtration rate (eGFR).

### Echocardiography

All patients underwent conventional transthoracic echocardiography at the time of diagnosis. Cardiac chamber sizes were quantified according to previous recommendations from current guidelines^24^. Mean left ventricular (LV) wall thickness was derived from the average of posterior left ventricular (LV) wall and interventricular wall thickness at end-diastole. The ratio of early transmitral flow to early septal mitral annular diastolic velocity (E/e′) was measured as an index of LV filling pressure^25^.

### Biomarkers

NT-proBNP was measured with an electrochemiluminescence sandwich immunoassay (Elecsys system 2010^®^, Roche Diagnostics, Mannheim, Germany). TnT assay was performed with a fourth generation assay (Elecsys 2010^®^, Roche Diagnostics, Mannheim, Germany).

Blood samples were frozen immediately and stored at −70 °C before measurement of sST2, GDF-15, and OPN. Plasma OPN levels were measured with a sandwich immunoassay by use of a commercially available kit (Quantikine^®^; R&D Systems, Inc., Minneapolis, MN, USA). Plasma levels of soluble suppression of tumorigenicity 2 (sST2) were determined using a novel high-sensitivity sandwich immunoassay (Presage ST2^®^; Critical Diagnostics, San Diego, CA, USA). Plasma levels of growth differentiation factor 15 (GDF15) were determined by a sandwich enzyme immunoassay technique (Quantikine^®^; R&D Systems, Inc., Minneapolis, MN, USA).

### Statistical analysis

Continuous data were expressed as mean ± standard deviation and categorical variables were expressed as absolute number (percent). Receiver operating characteristics (ROC) curves were used to assess the predictive accuracy of biomarkers on 1-year all-cause mortality. The incremental prognostic utility of sST2 and GDF-15 was assessed by comparing the global chi square values. For comparison among three groups, which were classified according to sST2 and GDF-15 levels, one way ANOVA (parametric) and Kruskal-Wallis test (non-parametric) were used. Difference in overall survival was assessed using log-rank analysis with right censoring and displayed by Kaplan-Meier survival curve. Cox regression analysis was performed to investigate independent parameters to predict overall survival rates. All analyses were performed using IBM SPSS Statistics version 24 (SPSS Inc., Chicago, IL, USA).

### Statement

Informed consent was obtained from all subjects, and all methods were carried out in accordance with the relevant guidelines and regulations according to the principles expressed in the Declaration of Helsinki. All study protocols were approved by Institutional Review Board at Samsung Medical Center.

### Disclosures

This research was supported by a grant (code: 2016E6300502) from the Research of Korea Centers for Disease Control and Prevention

## Supplementary information

supplementary information

## Data Availability

The datasets generated during and/or analyzed during the current study are available from the corresponding author on reasonable request.
