# Supplementary material for: Prognostic values of novel biomarkers in patients with AL amyloidosis
Source: Sci Rep. 2019 Aug 21;9:12200. doi: 10.1038/s41598-019-48513-6 (PMC6704139; doi:10.1038/s41598-019-48513-6)
Supplement: Supplementary file 1 — supplementary information [file 41598_2019_48513_MOESM1_ESM.pdf]

Prognostic values of novel biomarkers in patients with AL amyloidosis

Darae Kim, MD, PhD[1], Ga Yeon Lee, MD[1], Jin-Oh Choi, MD, PhD[1], Kihyun Kim, MD, PhD[2], Seok Jin Kim MD, PhD[2], Eun-Seon Ju, BSc[1], Eun-Seok Jeon MD,PhD[1]

[1]Division of Cardiology, Department of Medicine, Heart Vascular Stroke Institute, Samsung Medical Center, Sungkyunkwan University School of Medicine, Seoul, Republic of Korea [2]Divsion of Hematology and Oncology, Department of Medicine, Samsung Medical Center, Sungkyunkwan University School of Medicine, Seoul, Republic of Korea

Supplemental Figure 1. Incremental predictive values of sST2 and GDF-15 over conventional biomarkers for predicting overall survival.

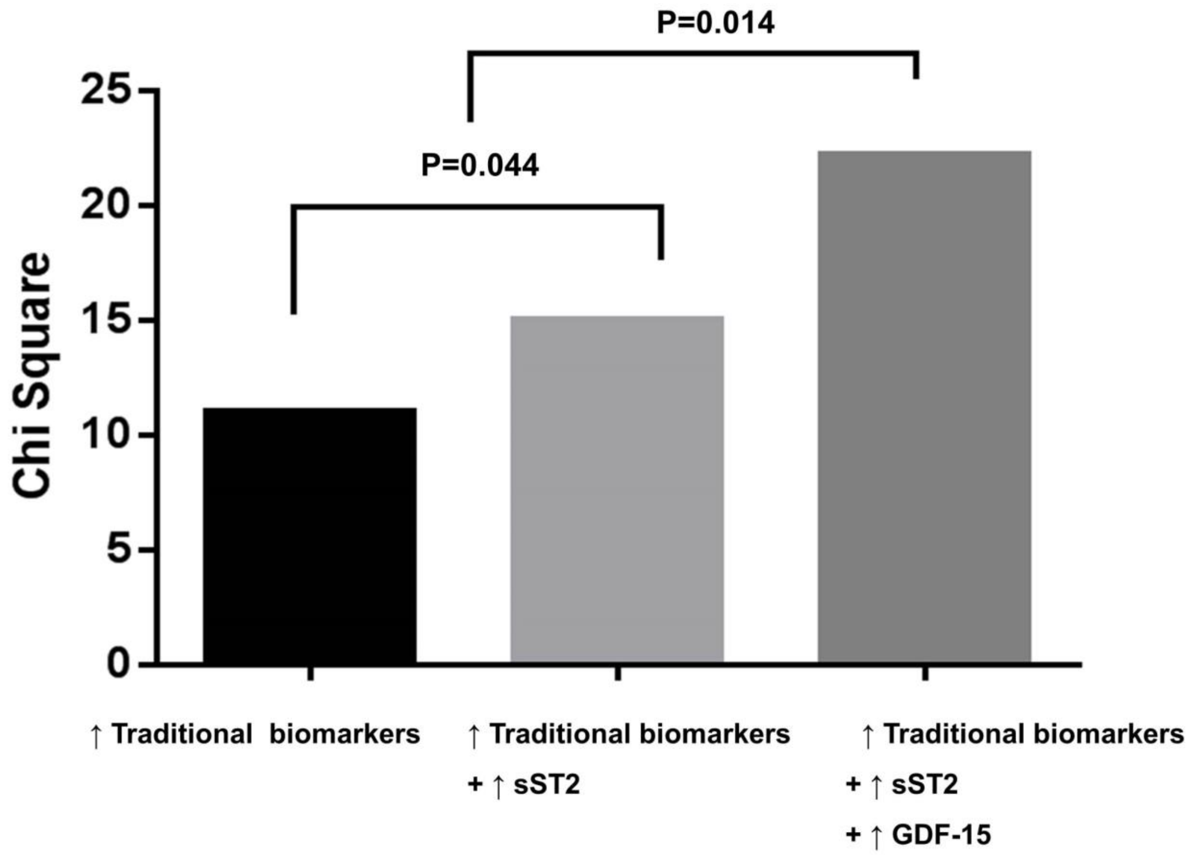

↑ Traditional biomarkers: TnT ≥ 0.025 ng/mL and/or NT-proBNP ≥ 1800 pg/mL

Supplemental Figure 2. Comparison of mean values of septal E' velocity (A) and mean wall thickness of left ventricle (B) according to scores given by levels of sST2 and GDF-15.

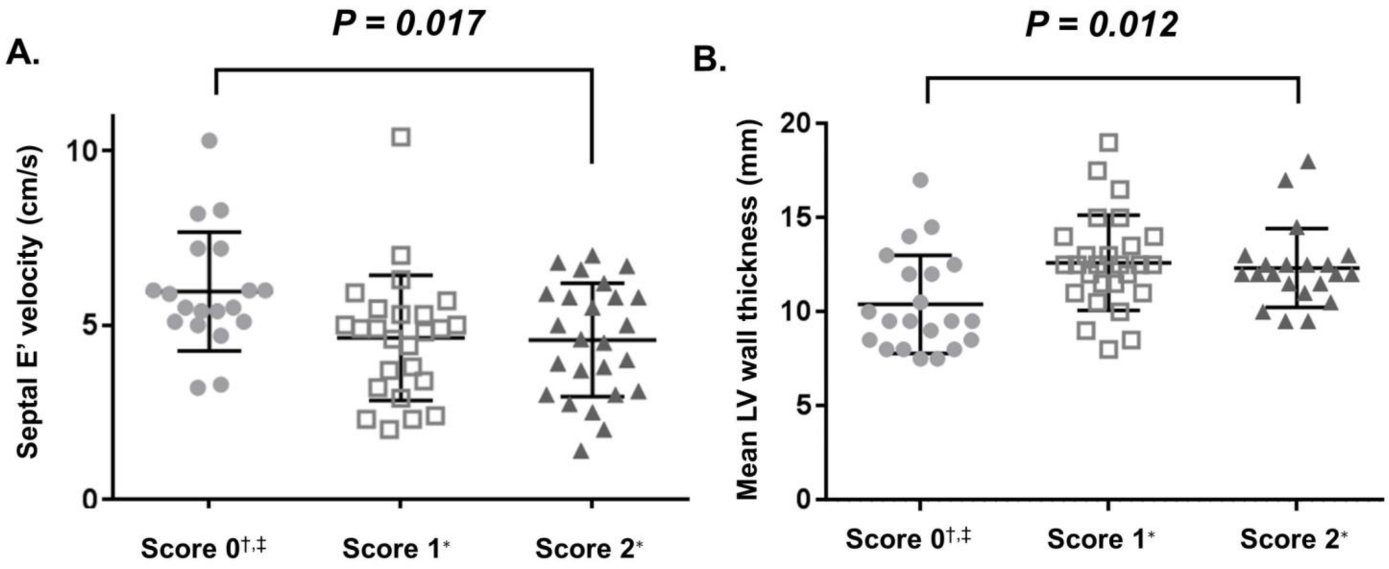

*, $p < 0.05$ when compared to S 0
†, $p < 0.05$ when compared to S 1
‡, $p < 0.05$ when compared to S 2

Supplemental Figure 3. Kaplan-Meier survival curve for overall survival according to scores given by levels of sST2 and GDF-15 and in subgroups of patients; patients with cardiac involvement (A) and with revised Mayo stages IV (B).

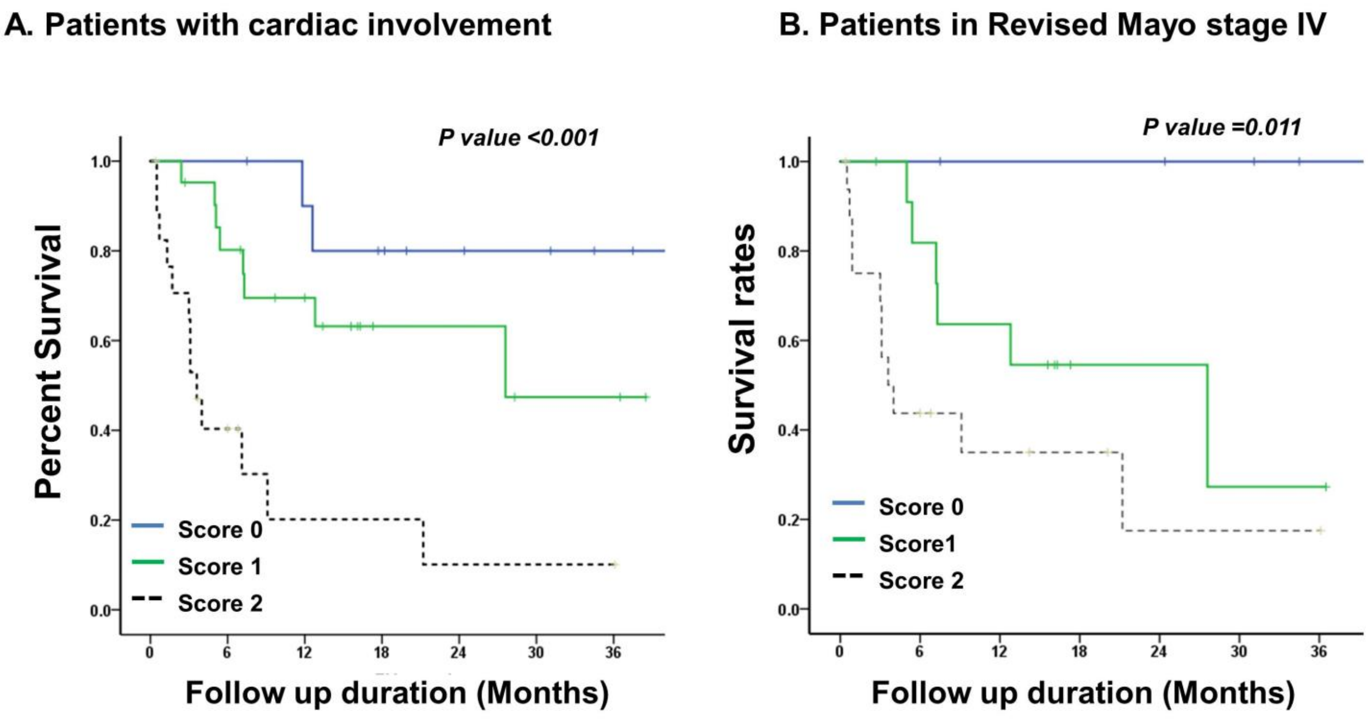

	0 M	6M	12M	18M	24M	36M
Total # at risk	50	29	22	12	9	4

	0 M	6M	12M	18M	24M	36M
Total # at risk	34	20	15	9	7	3
